# Efficacy and Safety of Topical Mechanistic Target of Rapamycin Inhibitors for Facial Angiofibromas in Patients with Tuberous Sclerosis Complex: A Systematic Review and Network Meta-Analysis

**DOI:** 10.3390/biomedicines10040826

**Published:** 2022-03-31

**Authors:** Yu-Ting Lin, Chia-Ling Yu, Yu-Kang Tu, Ching-Chi Chi

**Affiliations:** 1 Department of Pharmacy, Chang Gung Memorial Hospital, Linkou, Taoyuan 33305, Taiwan; anita851229@gmail.com (Y.-T.L.); beautyarielyou@gmail.com (C.-L.Y.); 2 Institute of Epidemiology and Preventive Medicine, College of Public Health, National Taiwan University, Taipei 10617, Taiwan; yukangtu@ntu.edu.tw; 3 Department of Dermatology, Chang Gung Memorial Hospital, Linkou, Taoyuan 33305, Taiwan; 4 College of Medicine, Chang Gung University, Taoyuan 33302, Taiwan

**Keywords:** angiofibroma, mechanistic target of rapamycin (mTOR), network meta-analysis, sirolimus, systematic review, tuberous sclerosis complex

## Abstract

Previous studies have suggested that the topical mechanistic target of rapamycin (mTOR) inhibitors may be effective in treating facial angiofibromas in patients with tuberous sclerosis complex (TSC). Various concentrations of topical sirolimus for TSC have been tested, but their comparative efficacy and safety remained unclear. To assess the effects of topical mTOR inhibitors in treating facial angiofibromas, we conducted a systematic review and network meta-analysis (NMA) and searched MEDLINE, Embase, and Cochrane Library for relevant randomized controlled trials on 14 February 2022. The Cochrane Collaboration tool was used to assess the risk of bias of included trials. Our outcomes were clinical improvement and severe adverse events leading to withdrawal. We included three trials on 261 TSC patients with facial angiofibromas. The NMA found when compared with placebo, facial angiofibromas significantly improved following the application of various concentrations of topical sirolimus (risk ratio being 3.87, 2.70, 4.43, and 3.34 for 0.05%, 0.1%, 0.2%, and 1%, respectively). When compared with placebo, all concentrations of topical sirolimus did not differ in severe adverse events leading to withdrawal. The ranking analysis suggested topical sirolimus 0.2% as the most effective drug. In conclusion, topical sirolimus 0.05–1% are effective and safe in treating facial angiofibromas in patients with TSC, with topical sirolimus 0.2% being the most effective.

## 1. Introduction

Tuberous sclerosis complex (TSC) is an autosomal dominant disorder with an incidence of approximately 1 in 5000 to 10,000 live births [1]. TSC is characterized by abnormal skin pigmentation and tumor formation affecting multiple organs, causing benign tumors involving the skin, brain, kidney, and lung. TSC may also affect the central nervous system and cause seizures and neuropsychiatric disorders such as cognitive deficits and learning disabilities. In addition, there is an increased risk of malignancy in patients with TSC. The skin lesions of TSC appear in nearly all affected individuals and may serve as clues to diagnosis. Cutaneous manifestations of TSC may present with different types of skin lesions, including facial angiofibromas, hypomelanotic macules, fibrous cephalic plaques, shagreen patches, and periungual fibromas. Some skin findings, such as hypomelanotic macules, may appear at birth, whereas others, such as periungual fibromas, may not appear until adulthood. Facial angiofibromas generally appear between 2 and 5 years of age and occur in up to 74.5% to 90% of cases of TSC [2,3,4]. TSC results from mutations in either the TSC1 gene or the TSC2 gene [1,5,6]. The TSC1 gene, which maps to chromosome 9q34, spans 50 kb of genomic DNA and contains 23 exons. It encodes a 130 kDa protein called hamartin, which is widely expressed in normal tissues. Hamartin forms a complex with the tuberin protein that is encoded by the TSC2 gene. The TSC2 gene, which maps to chromosome 16p13.3, spans 45 kB of genomic DNA and contains 42 exons. The gene is ubiquitously expressed in all normal adult tissues, and encodes a 200 kDa protein, tuberin. The mechanistic target of rapamycin (mTOR), a conserved serine-threonine protein kinase, is present in the cytoplasm complexed with several other molecules. The main function of mTOR signaling is stimulation of protein synthesis, cell survival, and cell cycle progression through two distinct multimeric complexes: mTOR complex 1 and 2 (abbreviated as mTORC1 and mTORC2, respectively). In the nutrient-poor state, the TSC1–TSC2 complex inhibits activation of mTOR signaling, which prevents cell growth, protein synthesis, and cell division [7,8]. Complex involvement of TSC1 and TSC2 gene products in cell signaling has been demonstrated in the pathogenesis of TSC.

In patients with TSC, either the TSC1 gene or the TSC2 gene with pathogenic mutations inactivates the TSC protein complex. Sporadic TSC accounts for approximately two-thirds of all cases and family inheritance for approximately one-third. There is no difference in the prevalence of TSC between men and women. Some case reports or retrospective studies found that the contribution of TSC1 and TSC2 variants is similar in inherited genetic cases of TSC, while TSC2 mutations are four to five times more common in sporadic TSC cases. The main types of variants in TSC1 are point mutations and deletion/insertion of small fragments. Most of the variants in TSC2 are missense and nonsense mutations; less frequently, small/large fragment deletions; and splice site mutations, which are often accompanied by genetic recombination [6,9,10]. Pathogenic variants of either the TSC1 gene or the TSC2 gene lead to a loss of inhibitory effect on the mTOR pathway, which mediates cell growth and metabolism in response to alterations in growth factors, cellular energy, and nutrient status. TSC-associated tumors, including hamartomas, angiofibromas, and lymphangioleiomyomas, are characterized by the loss of heterozygosity [11,12]. The skin of TSC patients contains a mutant copy of either the TSC1 or TSC2 gene. A loss of heterozygosity results in a constitutive activation of mTOR with subsequent production of epidermal basal cells at a faster rate than the sloughing of the dead cells [13]. mTOR is activated in the proliferating fibroblast-like cells within facial angiofibromas. Cells with non-functional TSC genes also secrete vascular growth factors that induce angiogenesis. This overproduction of skin cells, in conjunction with angiogenesis, results in the formation of facial angiofibromas which contain plump, spindle-shaped, or stellate fibroblastic cells in the dermis among increased numbers of dilated vessels. An angiofibroma is a firm, flesh-colored, dome-shaped papule around 1 to 3 mm in diameter. It may be hyperpigmented, especially in individuals with darker pigmentation. They occur on the central face and are often aggregated in the alar grooves, extending symmetrically onto the cheeks and nose, nasal opening, and chin, with relative sparing of the upper lip and lateral face. The number of facial angiofibromas in a single patient ranges from 1 to more than 100. Lesions may coalesce to form large nodules, especially in the alar grooves. Sometimes, lesions occur on the forehead, scalp, or eyelids. These striking and visible skin lesions may cause substantial psychological distress to TSC patients. Multiple treatments for angiofibroma, including curettage, cryosurgery, chemical peels, dermabrasion, shave excisions, and laser therapy, have been developed to alleviate the appearance of these lesions [1]. Although most of these treatments are effective, they are invasive procedures and often need to be repeated at periodic intervals to treat the recurrence of facial angiofibromas. To date, there is no effective method for preventing or permanently removing facial angiofibromas in patients with TSC [3,14,15,16].

mTOR inhibitors, such as sirolimus or everolimus, bind with high specificity to mTOR, which results in inhibition of the hyperactivity of mTOR and ultimately in down-regulation of cell growth. Inhibition of the mTOR complex also results in decreased levels of vascular endothelial growth factor, thus depriving tumor cells of their vascular supply [17,18,19]. Sirolimus, also known as rapamycin, is a macrocyclic triene antibiotic that is produced by fermentation of *Streptomyces hygroscopicus* [20]. Everolimus is a derivative of sirolimus by the addition of an ethyl ester group and demonstrates better absorption, higher oral bioavailability, more rapid achievement of steady-state blood concentrations after administration, and faster elimination after withdrawal than sirolimus [8]. The U.S. Food and Drug Administration has approved oral everolimus for the treatment of renal and central nervous system involvement in TSC and oral sirolimus for treating lymphangioleiomyomatosis and renal allografts [8,21]. Favorable therapeutic results were obtained after clinical trials of oral sirolimus for treating TSC-related brain tumors, renal tumors, and lung lesions [18,22,23,24,25]. While long-term systemic administration of sirolimus is considered necessary for the maintenance of tumor regression, it may induce side effects such as infections or malignancies, stomatitis, mouth ulceration, acne-like skin lesions, hypertriglyceridemia, hypercholesterolemia, bone marrow suppression, proteinuria, joint pain, and noninfectious pneumonitis [17]. To avoid serious side effects from long-term systematic use of mTOR inhibitors, a topical mTOR inhibitor formulation was developed for the treatment of facial angiofibromas associated with TSC. There have been several trials illustrating the clinical efficacy of topical mTOR inhibitors for the treatment of incurable facial angiofibroma associated with TSC [17,24,26,27,28,29]. However, the concentrations of topical sirolimus differed among these trials, ranging from 0.003% to 1%. In this study, we aimed to ascertain which concentration of topical sirolimus is the best in clinical efficacy and safety for the treatment of facial angiofibromas associated with TSC.

## 2. Methods

We conducted a systematic review and network meta-analysis (NMA) of randomized controlled trials (RCTs) that examined the effects of different concentrations of topical sirolimus in treating facial angiofibromas in patients with TSC. The reporting of this study followed the Preferred Reporting Items for Systematic Reviews and Meta-Analyses for Network Meta-Analyses (PRISMA-NMA) extension [30,31]. We have registered our protocol with PROSPERO (CRD42021228510; see https://www.crd.york.ac.uk/prospero/display_record.php?ID=CRD42021228510 (accessed on 8 February 2021)). 

### 2.1. Evidence Searches

We searched MEDLINE, Embase, and Cochrane Central Register of Controlled Trials (CENTRAL) databases for relevant studies until 14 February 2022. The search terms included ‘facial angiofibroma’, ‘tuberous sclerosis complex’, mTOR inhibitors, and their specific generic names. The search strategy is listed in Table S1 (see Supplementary Materials). No limitations on language or geographic locations were applied.

### 2.2. Selection of Studies

Two authors (Y.L. and C.Y.) independently selected relevant studies based on the following inclusion criteria: (1) the study design was RCT; (2) the participants were TSC patients with facial angiofibroma; and (3) the study medication was a topical mTOR inhibitor. Studies were excluded if (1) there were no control groups for comparison, (2) they were case reports or case series, and (3) they lacked usable data. We evaluated the titles and abstracts of the retrieved literature. If the abstract did not provide enough information to include or exclude the study, we evaluated the full text to determine the eligibility. Discrepancies in study selection were resolved by discussion with a senior author (C.C.). Our outcomes of interest were (1) clinical improvement of facial angiofibromas (overall and in terms of erythema and size) and (2) severe adverse events leading to withdrawal of treatment. 

### 2.3. Data Extraction and Risk of Bias Assessment 

We extracted the data in a predefined spreadsheet. The extracted data from each study were publication year, setting (study population and country), study details (intervention and follow-up period), and the number of participants. Two authors (Y.L. and C.Y.) independently utilized the Cochrane Collaboration tool to assess the risk of bias of included RCTs [32]. Any unresolved discrepancies in the data extraction or appraisal of the results were resolved by discussion with a third author (C.C.).

### 2.4. Statistical Analysis 

We conducted NMA to combine direct and indirect evidence. Because the number of included trials per comparison was few, the fixed-effect model was chosen [33]. We calculated the risk ratio (RR) with a 95% confidence interval (CI) of each intervention compared with placebo for both outcomes [34,35].

We calculated the relative ranking probabilities for the interventions and obtained the surface under the cumulative ranking curve (SUCRA) as the percentage of the mean rank of each intervention [36]. The higher the SUCRA value, and the closer to 100%, the higher the likelihood that a therapy is in the top rank or one of the top ranks [37]. We drew a two-dimensional ranking plot incorporating the two outcomes of interest, i.e., clinical improvement and severe adverse events leading to withdrawal. Treatments in the upper right corner in the plot were more effective and safer than the other treatments [38,39]. Due to the small number of included RCTs in our NMA, funnel plots and Egger’s test were not performed to assess publication bias. All statistical analyses were performed by using the NMA suite for the Stata version 15.1 (StataCorp. 2017. Stata Statistical Software: Release 15. College Station, TX: StataCorp LLC. Texas, USA) [40].

## 3. Results

### 3.1. Study Selection 

**Figure 1:**
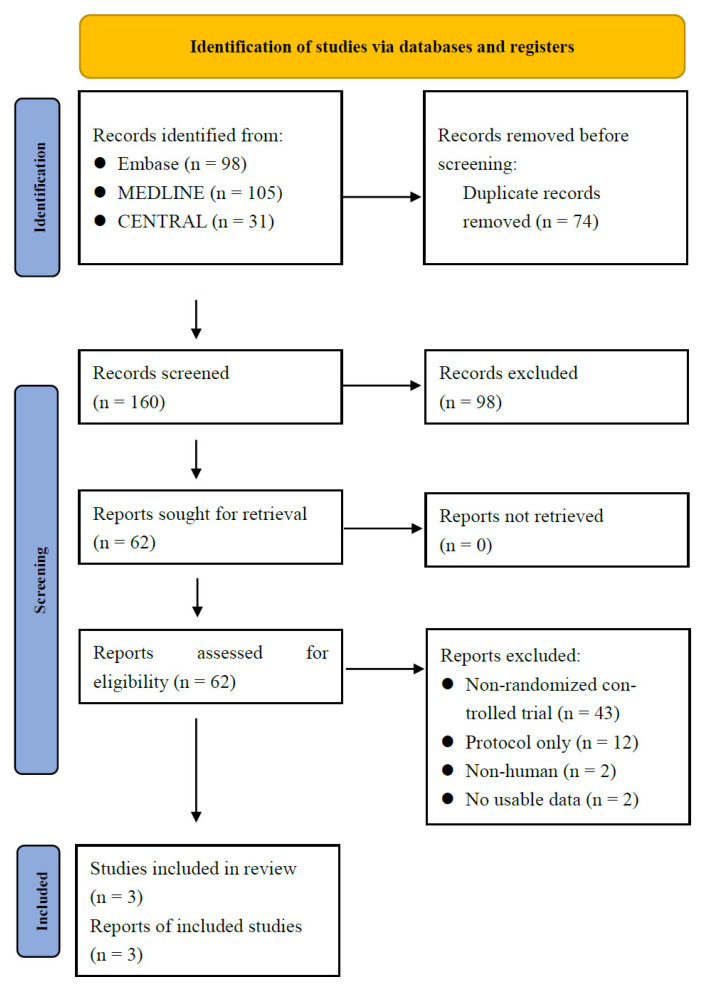
PRISMA study flow chart. CENTRAL, Cochrane Central Register of Controlled Trials.

As illustrated in the PRISMA study flow chart (Figure 1), 228 records were identified through database searching (MEDLINE = 102; Embase = 95; CENTRAL = 31). After removing duplicates, 160 records were screened by title and abstract, yielding 62 articles for full-text assessment. Finally, there were five RCTs included in this study. We excluded two RCTs for the following reasons. One study was excluded due to the lack of respective data for individual concentrations of topical sirolimus [27]. Another trial was a double-blind spilt-face RCT that compared topical rapamycin 0.1% or calcitriol 0.0003% single-agent therapy; however, there was a lack of data on the effects of topical sirolimus alone [29]. Eventually, there were three studies with 261 TSC patients with facial angiofibroma included in this NMA [24,26,41].

### 3.2. Characteristic and Risk of Bias of Included Studies 

The characteristics of the included RCTs are summarized in Table 1. The three included studies were phase II or III RCTs completed between 2012 and 2016. The risk of bias of the three included studies was generally low (see Figure S1 in the Supplementary Materials).

### 3.3. Clinical Improvement

#### 3.3.1. Overall Geometric Structure of the Whole Network

**Figure 2:**
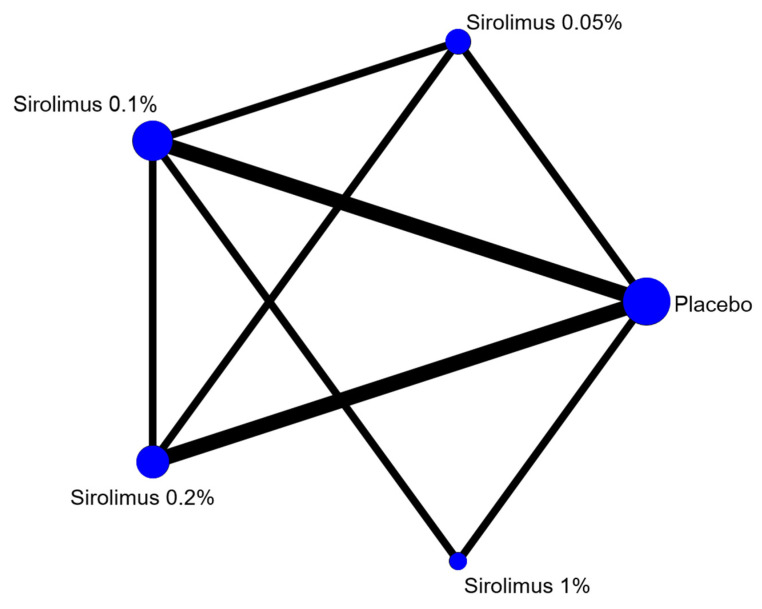
Geometry of comparisons in the network meta-analysis.

NMA was performed to compare the effects of different concentrations of topical sirolimus in treating facial angiofibromas. The geometry of the network is presented in Figure 2. Four concentrations (0.05%, 0.1%, 0.2%, and 1%) of topical sirolimus were included in the network.

#### 3.3.2. Network Meta-Analysis for Efficacy Evaluation

**Figure 3:**
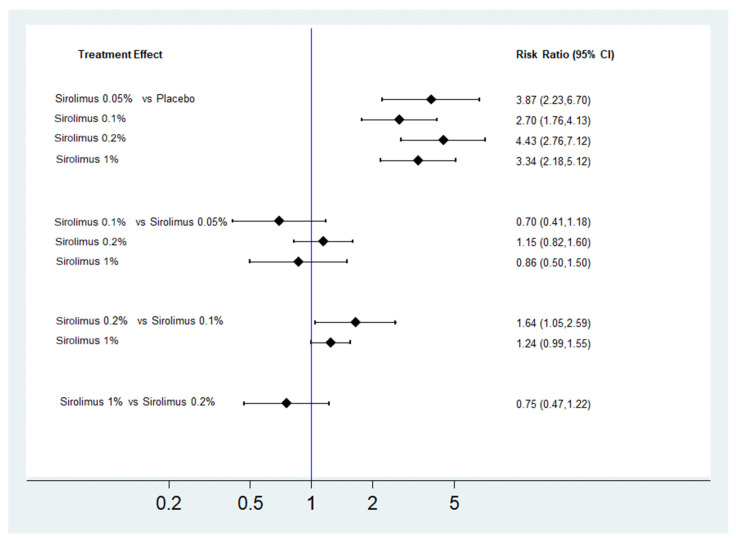
Forest plots of network meta-analysis of treatments for facial angiofibromas: analysis based on clinical improvement.

As shown in Figure 3, the NMA illustrated that topical sirolimus 0.05%, 0.1%, 0.2%, and 1% provided significant clinical improvement when compared with placebo (RR = 3.87, 95% CI = 2.23–6.7 for topical sirolimus 0.05%; RR = 2.70, 95% CI = 1.76–4.13 for topical sirolimus 0.1%; RR = 4.43, 95% CI = 2.76–7.12 for topical sirolimus 0.2%; and RR = 3.34, 95% CI = 2.18–5.12 for topical sirolimus 1%). Topical sirolimus 0.2% showed significantly superior effects in clinical improvement when compared with topical sirolimus 0.1% (RR = 1.64, 95% CI = 1.05–2.59).

#### 3.3.3. Surface under the Cumulative Ranking Curve for Efficacy Evaluation

Regarding the ranking in treatment efficacy (see Table S3 in the Supplementary Materials), topical sirolimus 0.2% ranked the best in clinical improvement (SUCRA = 90.8%), followed by topical sirolimus 0.05% (SUCRA = 70.4%), topical sirolimus 1% (SUCRA = 60.2%), and topical sirolimus 0.1% (SUCRA = 28.6%).

### 3.4. Severe Adverse Events Leading to Withdrawal

#### 3.4.1. Network Meta-Analysis for Safety Evaluation 

The NMA on severe adverse events leading to withdrawal of treatment found no significant differences between different concentrations of topical sirolimus and placebo (RR = 0.99, 95% CI = 0.83–1.18 for topical sirolimus 0.05%; RR = 1, 95% CI = 0.97–1.04 for topical sirolimus 0.1%; RR = 1, 95% CI = 0.94–1.06 for topical sirolimus 0.2%; and RR = 0.98, 95% CI = 0.93–1.03 for topical sirolimus 1%, see Figure S2 in the Supplementary Materials).

#### 3.4.2. Surface under Cumulative Ranking Curve for Safety Evaluation

The ranking based on severe adverse events leading to withdrawal was as follows: topical sirolimus 0.05% (SUCRA = 47.4%), topical sirolimus 0.1% (SUCRA = 60%), topical sirolimus 0.2% (SUCRA = 53.5%), and topical sirolimus 1% (SUCRA = 31.6%) (see Table S4 in the Supplementary Materials).

### 3.5. Ranking Plot Analysis of Different Treatments

**Figure 4:**
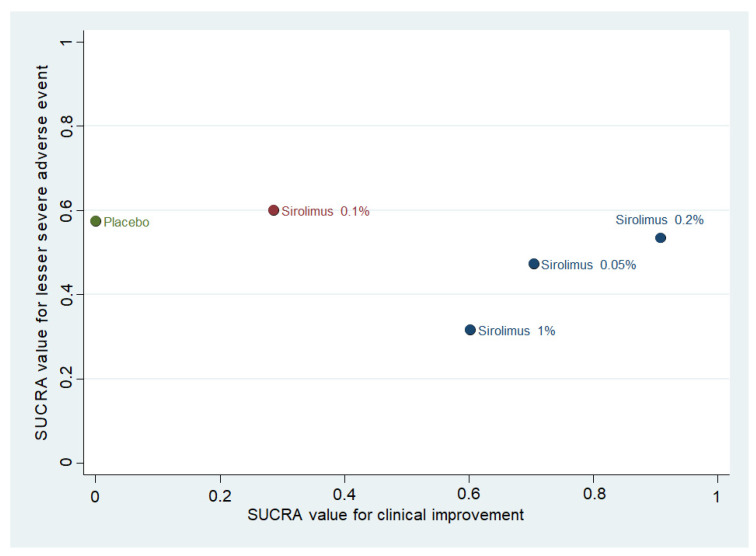
Ranking plot of different treatments for facial angiofibroma based on the surface under the cumulative ranking curve (SUCRA) values for two outcomes: clinical improvement and severe adverse events leading to withdrawal. Treatments in the upper right corner were associated with greater increase in complete clearance and lesser adverse events associated withdrawal than the other treatments.

The ranking plot based on the SUCRA values for clinical improvement and severe adverse events leading to withdrawal is shown in Figure 4. Topical sirolimus 0.2% was the drug associated with the best efficacy, while the safety profile of all interventions did not differ substantially.

### 3.6. Publication Bias

Funnel plots and Egger’s test were not performed to assess publication bias owing to the small number of included RCTs in each comparison.

## 4. Discussion

Our NMA suggests topical sirolimus 0.2% as the most effective in terms of clinical improvement (Figure 4). Our study illustrated that different concentrations of topical sirolimus were superior to placebo in clinical improvement (RR = 3.87, 95% CI = 2.23–6.7 for 0.05%; RR = 2.70, 95% CI = 1.76–4.13 for 0.1%; RR = 4.43, 95% CI = 2.76–7.12 for 0.2%; and RR = 3.34, 95% CI = 2.18–5.12 for 1%). On the other hand, all concentrations of topical sirolimus were generally well tolerated. Minor side effects such as irritation, burning, pruritus, and a dry sensation at application site were the most common adverse events seen after topical sirolimus [24,26,41]. One RCT included in our analysis reported one patient receiving topical sirolimus 1% who withdrew topical sirolimus due to mild facial cutaneous eruption around the application site [26]. The other two RCTs reported no medication-related withdrawals [24,41].

TSC is a genetic multisystem disorder with mutations of TSC1 and TSC2 genes, leading to a loss of inhibitory effect on the mTOR pathway, then resulting in cell overgrowth and eventually causing widespread hamartomatous tumors in several organs. mTOR inhibitors bind to the mTOR complex at allosteric sites that restores metabolic homeostasis in abnormal cells, thereby reversing TSC-associated clinical manifestations. Oral ingestion of mTOR inhibitors such as sirolimus and everolimus have been used to induce the regression of TSC-related tumors, and both compounds bind to mTORC1 [7,21,42,43]. Following entry into the cytoplasm, sirolimus and everolimus bind to the FK-binding protein-12 (FKBP-12) and presumably modulate the activity of the mTOR. The mTOR inhibitors abort IL-mediated signal transduction and result in T and B cell cycle arrest in the G1-S phase. Sirolimus and everolimus block the response of T and B cell activation by cytokines, which prevents cell cycle progression and proliferation [44,45]. Most patients treated with an oral mTOR inhibitor for internal tumors also show improvement in their skin lesions. Therefore, mTOR inhibitors may be candidates for alleviating TSC-related skin manifestations. In 2012, the first study of topical sirolimus for the treatment of TSC-associated facial angiofibromas demonstrated considerable efficacy and safety in 28 patients aged > 13 years (blood concentrations of sirolimus was lee than 1.0 ng/mL). In the study, 73% of patients treated with topical sirolimus versus 38% of patients treated with placebo reported a subjective improvement in angiofibromas [27]. In recent years, there have been more references (RCTs, case reports, and retrospectives studies) demonstrating that the skin lesions of TSC could be effectively treated with topical mTOR inhibitors, especially sirolimus, which is the oldest mTOR inhibitor, with a well-known safety profile [12,28]. Regarding severe adverse events leading to withdrawal, our NMA found no differences between placebo and various concentrations of topical sirolimus. The safety of topical sirolimus may be attributed to its minimal systemic absorption. The serum levels of sirolimus among the participants receiving topical sirolimus were low or undetectable (0.2 ± 0.1 ng/mL), while the therapeutic levels of systemic sirolimus were 5–15 ng/mL [24,26,41,46].

There were limitations in the current available evidence. First, there were variations in the preparations of topical sirolimus because there were no commercially available preparations in the studies we included. However, since 2018, there has been one commercial product: RAPALIMUS^®^ in Japan, which mainly comprises sirolimus 0.2% topical gel. Second, there were only three RCTs available in our study. More large-scale RCTs are warranted to verify our findings and test if other mTOR inhibitors work. Third, two included RCTs assessed overall improvement based on the investigators’ evaluation, whereas another RCT evaluated composite improvement in size and color in facial angiofibromas. The different methods of measuring clinical improvement might have introduced methodological heterogeneity. Therefore, the results of this study should be interpreted carefully. There are reported tools for the efficacy of treatment to be reliably and reproducibly evaluated for treating facial angiofibromas. The Facial Angiofibroma Severity Index (FASI) is evaluated by the grade of erythema and the size and extent of the cheek surface of facial angiofibromas, assessing each of the categories on a four-point scale (0–3) [47]. Modified FASI (mFASI) is evaluated by the grade of erythema, size, papule elevation, and extent of lesions, assessing each of the categories on a four-point scale (0–3) [29]. The Angiofibroma Grading Scale (AGS) scores is evaluated by erythema, lesion density, average lesion size, and percent involvement for facial angiofibromas, assessing each of the categories on a five-point scale (0–4) [26]. Fourth, different vehicles such as creams and gels have been used in the included trials, which might have various lipophilicities affecting the bioavailability of sirolimus [48,49,50].

## 5. Conclusions

Topical sirolimus 0.05–1% is an effective and safe treatment of facial angiofibromas in patients with TCS. We identified topical sirolimus 0.2% as the drug associated with the best efficacy, while the safety profile of various concentrations of sirolimus did not differ substantially. Based on the current limited evidence, 0.2% topical sirolimus may be an optimal treatment option for facial angiofibromas in patients with TSC. 

## Figures and Tables

**Table 1:** Characteristics and included studies.

First Author, Year, Country	Participants	Interventions	Outcomes
Wataya-Kaneda, 2017, [41] Japan Settings: 1 center	Inclusion: Age: 6–47 years Diagnosis: TSC and ≥3 isolated facial angiofibromas (≥2 mm in diameter) Exclusion: mTOR inhibitors within the past 12 months Surgical treatments (including laser) within the past 6 months Topical tacrolimus within the past 3 months	Randomization carried out by 2:1 to the following: Placebo twice daily for 12 weeks (*n* = 12) 0.05% sirolimus gel twice daily for 12 weeks (*n* = 8) 0.1% sirolimus gel twice daily for 12 weeks (*n* = 8) 0.2% sirolimus gel twice daily for 12 weeks (*n* = 8)	Ratio for the decrease in tumor volume (weeks 2, 4, 8, 12, 16) Reduction in tumor color (weeks 2, 4, 8, 12, 16) Improvement factor (variable composed of tumor volume reduction and lessening of the redness of the 3 target tumors) (weeks 2, 4, 8, 12, 16) Photography (week 12) General improvement (week 12) Adverse event (no. of AEs): Dry skin (placebo, *n* = 1; 0.05%, *n* = 4; 0.1%, *n* = 3; 0.2%, *n* = 5) Irritated skin (placebo, *n* = 3; 0.05%, *n* = 2; 0.1%, *n* = 2; 0.2%, *n* = 4)
Koenig, 2018, [26] USA Settings: 9 clinical sites in the USA and 1 in Australia	Inclusion criteria: Age: 3–61 years Diagnosis: TSC and visible facial angiofibromas Exclusion: Sirolimus or immunosuppression receiverb Immune dysfunction or oral mTOR inhibitor receiverc Pregnant or nursingd Dermatologic condition that could interfere with study assessmentse Hypersensitivity to the topical formulation or sirolimusf Dermatologic treatment for their facial angiofibromas within the past 6 monthsg Participation in clinical trial within the past 30 days	Randomization carried out by 1:1:1 to the following Placebo every evening for 6 months (*n* = 57) 0.1% sirolimus gel every evening for 6 months (*n* = 63) 1% sirolimus gel every evening for 6 months (*n* = 59)	Change from baseline in the angiofibroma grading scale (in 6 months) Photo readers’ rating of paired baseline and end of trial photographs for each patient (in 6 months) Adverse event (no. of AEs): Irritated skin (placebo, *n* = 0; 0.1%, *n* = 2; 1%, *n* = 1)
Wataya-Kaneda, 2018, [24] Japan Settings: multi-center (9 sites)	Inclusion: Age: 3 years and older Diagnosis: TSC and ≥3 reddish papules facial angiofibromas (≥2 mm in diameter) Exclusion: Erosions, ulcers, or other skin lesions associated with angiofibromas Inadequately photographed skin lesions Significant comorbidities including poorly controlled dyslipidemia mTOR inhibitor within 12 months prior to use of the investigational drug Laser therapy or surgery within 6 months	Randomization carried out by 1:1 to the following: Placebo twice daily for 12 weeks (*n* = 32) 0.2% sirolimus gel twice daily for 12 weeks (*n* = 30)	Composite improvement in angiofibromas based on the efficacy variables (size and color) (weeks 4, 8, 12, 16) Adverse event (no. of AEs): Dry skin (placebo, *n* = 4; 0.2%, *n* = 11) Irritated skin (placebo, *n* = 9; 0.2%, *n* = 11)

Abbreviations: TSC: tuberous sclerosis complex; mTOR: mechanistic target of rapamycin.

## Data Availability

Data collected and extracted into Excel sheets used in our analyses are available upon reasonable request from the corresponding author.

## Supplementary Materials

The following supporting information can be downloaded at: https://www.mdpi.com/article/10.3390/biomedicines10040826/s1, Table S1: Search strategy. Table S2: List of excluded studies after full-text screening. Table S3: Surface under the cumulative ranking curve (SUCRA) of clinical improvement. Table S4: Surface under the cumulative ranking curve (SUCRA) of treatments based on severe adverse events leading to withdrawal. Figure S1: Summary for risk of bias of included clinical trials. The green symbols represent low risk of bias, the yellow symbols represent unclear risk of bias, and the red symbols represent high risk of bias. Figure S2: Forest plots of network meta-analysis of treatments for facial angiofibroma: analysis based on severe adverse events leading to withdrawal. Figure S3: Clinical improvement. Forest plots from pairwise comparisons of treatment agents. The result of a single study was identified as direct evidence when pairwise meta-analysis for a specific comparison was not attainable. Figure S4: Severe adverse events leading to withdrawal. Forest plots from pairwise comparisons of treatment agents. The result of a single study was identified as direct evidence when pairwise meta-analysis for a specific comparison was not attainable.
