# Supplementary material for: Efficacy and Safety of Topical Mechanistic Target of Rapamycin Inhibitors for Facial Angiofibromas in Patients with Tuberous Sclerosis Complex: A Systematic Review and Network Meta-Analysis
Source: Biomedicines. 2022 Mar 31;10(4):826. doi: 10.3390/biomedicines10040826 (PMC9025300; doi:10.3390/biomedicines10040826)

Supplementary Materials

Yu-Ting Lin, Chia-Ling Yu, Yu-Kang Tu and Ching-Chi Chi

Efficacy and safety of topical mechanistic target of rapamycin inhibitors for facial angiofibromas in patients with tuberous sclerosis complex: a systematic review and network meta-analysis.

Supplementary Information

List of supplementary materials

Table S1 Search strategy

Table S2 List of excluded studies after full-text screening

Table S3 Surface under the cumulative ranking curve (SUCRA) of clinical improvement

Table S4 Surface under the cumulative ranking curve (SUCRA) of treatments based on severe adverse events leading to withdrawal

Figure S1 Summary for risk of bias of included clinical trials. The green symbols represent low risk of bias, the yellow symbols represent unclear risk of bias, and the red symbols represent high risk of bias

Figure S2 Forest plots of network meta-analysis of treatments for facial angiofibroma: analysis based on severe adverse events leading to withdrawal

Figure S3 Clinical improvement

Forest plots from pairwise comparisons of treatment agents. The result of a single study was identified as direct evidence when pairwise meta-analysis for a specific comparison was not attainable.

Figure S4 Severe adverse events leading to withdrawal

Forest plots from pairwise comparisons of treatment agents. The result of a single study was identified as direct evidence when pairwise meta-analysis for a specific comparison was not attainable.

Table S1 Search strategy

**MEDLINE search strategy**
#1 exp angiofibroma / or angiofibroma.mp.
#2 exp tuberous sclerosis / or tuberous sclerosis.mp.
#3 mTOR inhibitor.mp.
#4 sirolimus.mp.
#5 rapamycin.mp.
#6 everolimus.mp.
#7 temsirolimus.mp.
#8 deforolimus.mp.
#9 Ridaforolimus.mp.
#10 (#1 and #2)
#11 (#3 OR #4 OR #5 OR #6 OR #7 OR #8 OR #9)
**CENTRAL search strategy**
#1 ("angiofibroma "):ti,ab,kw
#2 MeSH descriptor: [angiofibroma] explode all trees
#3 #1 or #2 `
#4 ("tuberous sclerosis "):ti,ab,kw
#5 MeSH descriptor: [tuberous sclerosis] explode all trees
#6 #4 OR #5
#7 #3 and #6
#8 ("mTOR inhibitor "):ti,ab,kw
#9 ("sirolimus "):ti,ab,kw
#10 ("rapamycin"):ti,ab,kw
#11 ("everolimus "):ti,ab,kw
#12 ("temsirolimus "):ti,ab,kw
#13 ("deforolimus "):ti,ab,kw
#14 ("Ridaforolimus "):ti,ab,kw
#15 (#8 OR #9 OR #10 OR #11 OR #12 OR #13 OR #14)
#16 (#7and #14)
**EMBASE search strategy**
#1 exp angiofibroma / or angiofibroma.mp.
#2 exp tuberous sclerosis / or tuberous sclerosis.mp.
#3 mTOR inhibitor.mp.
#4 sirolimus.mp.
#5 rapamycin.mp.
#6 everolimus.mp.
#7. temsirolimus.mp.
#8. deforolimus.mp.
#9 Ridaforolimus.mp.
#10 (#1 and #2)
#11 (#3 OR #4 OR #5 OR #6 OR #7 OR #8 OR #9)
#12 (#10 and #11)

CENTRAL: Cochrane Central Register of Controlled Trials

Table S2 List of excluded studies after full-text screening

First author	Title	Citation	Exclusion reason
**Leducq, S.**	Topical use of mammalian target of rapamycin inhibitors in dermatology: A systematic review with meta-analysis	Journal of the American Academy of Dermatology 2019;80:735-42.	Case report
**Tanaka, M.**	First left-right comparative study of topical rapamycin vs. vehicle for facial angiofibromas in patients with tuberous sclerosis complex	British Journal of Dermatology 2013;169:1314-8.	Case report
**Spalgais, S.**	Pulmonary choriostoma in a case of tuberous sclerosis complex	Journal of Postgraduate Medicine 2015;61:193-6.	Case report
**Wheless JW**	A novel topical rapamycin cream for the treatment of facial angiofibromas in tuberous sclerosis complex.	Journal of Child Neurology 2013;28:933-6.	Case report
**Wataya-Kaneda M**	A topical combination of rapamycin and tacrolimus for the treatment of angiofibroma due to tuberous sclerosis complex (TSC): a pilot study of nine Japanese patients with TSC of different disease severity.	British Journal of Dermatology 2011;165:912-6.	Case report
**Valeron-Almazan**	Topical rapamycin solution to treat multiple facial angiofibromas in a patient with tuberous sclerosis].	Actas Dermo-Sifiliograficas 2012;103:165-6.	Case report
**Tiedemann Svendsen M**	Facial angiofibromas associated to tuberous sclerosis treated with topical sirolimus.	Ugeskr Laeger 2013;175:2569-70.	Case report
**Salido R**	Sustained clinical effectiveness and favorable safety profile of topical sirolimus for tuberous sclerosis - associated facial angiofibroma.	J Eur Acad Dermatol Venereol 2012;26:1315-8	Case report
**Mutizwa MM**	Treatment of facial angiofibromas with topical application of oral rapamycin solution (1mgmL(-1)) in two patients with tuberous sclerosis.	British Journal of Dermatology 2011;165:922-3.	Case report
**Mackel F**	Does the mTOR inhibitor everolimus influence the course of epilepsy in children with tuberous sclerosis complex?	Neuropediatrics. 2013;44(2).	Case report
**Kaufman McNamara E**	Successful treatment of angiofibromata of tuberous sclerosis complex with rapamycin.	Journal of Dermatological Treatment. 2012;23(1):46-48.	Case report
**Hwang SR**	Topical rapamycin for treating facial angiofibroma of tuberous sclerosis.	Journal of Dermatology. 2012;39:196.	Case report
**Haemel AK**	Topical rapamycin: a novel approach to facial angiofibromas in tuberous sclerosis.	Archives of Dermatology. 2010;146 (7):715-718.	Case report
**Foster RS**	Topical 0.1% rapamycin for angiofibromas in paediatric patients with tuberous sclerosis: a pilot study of four patients.	Australasian Journal of Dermatology.2012;53 (1):52-56.	Case report
**Dill PE**	Topical everolimus for facial angiofibromas in the tuberous sclerosis complex. A first case report.	Pediatric Neurology. 2014;51(1):109-113.	Case report

**DeKlotz CM**	Dramatic improvement of facial angiofibromas in tuberous sclerosis with topical rapamycin: optimizing a treatment protocol.	Archives of Dermatology. 2011;147(9):1116-1117.	Case report
**Cuevas Asencio I**	Topical sirolimus 0.4% formulation for treatment of facial angiofibromas.	Farm. 2012;36(5):433-434.	Case report
**Bloemen-Boot MCT**	Topical rapamycin for the treatment of facial angiofibromas with tuberous sclerosis complex.	Nederlands Tijdschrift Voor Dermatologie en Venereologie.2014;24(5):323-326.	Case report
**Wheless MC**	Long-Term Exposure and Safety of a Novel Topical Rapamycin Cream for the Treatment of Facial Angiofibromas in Tuberous Sclerosis Complex: Results From a Single-Center, Open-Label Trial.	Child Neurology Open. 2019;6.	Case report
**Wataya-Kaneda M**	Safety and Efficacy of the Sirolimus Gel for TSC Patients With Facial Skin Lesions in a Long-Term, Open-Label, Extension, Uncontrolled Clinical Trial.	Dermatology and therapy.2020;10(4):635-650.	Case report
**Wang S**	Tuberous Sclerosis Complex in 29 Children: Clinical and Genetic Analysis and Facial Angiofibroma Responses to Topical Sirolimus.	Pediatric Dermatology. 2017;34(5):572-577.	Case report
**Vasani RJ**	Facial angiofibromas of tuberous sclerosis treated with topical sirolimus in an Indian patient.	Indian Journal of Dermatology. 2015;60(2):165-169.	Case report
**Tu J**	Topical rapamycin for angiofibromas in paediatric patients with tuberous sclerosis: follow up of a pilot study and promising future directions.	Australasian Journal of Dermatology. 2014;55(1):63-69.	Case report
**Schwartz RA**	Facial angiofibromas of tuberous sclerosis treated with topical sirolimus in an Indian patient (commentary on article by Resham J. Vasani).	Dermatologic Therapy. 2016;29(1):70-71.	Case report
**Samanta D**	Topical mTOR (mechanistic target of rapamycin) inhibitor therapy in facial angiofibroma. Indian Journal of	Venereology and Leprology.2015;81(5):540-541.	Case report

	Dermatology,		
**Safa G**	Topical rapamycin for facial angiofibromas in tuberous sclerosis complex.	Oxford Medical Case Reports.2017;2017(7): 104-105.	Case report
**Pynn EV**	Successful topical rapamycin treatment for facial angiofibromata in two children.	Pediatric Dermatology. 2015;32(3):e120-123.	Case report
**Park J**	Treatment of angiofibromas in tuberous sclerosis complex: the effect of topical rapamycin and concomitant laser therapy.	Dermatology.2014;228 (1):37-41.	Case report
**Okanishi T**	Early Sirolimus Gel Treatment May Diminish Angiofibromas and Prevent Angiofibroma Recurrence in Children With Tuberous Sclerosis Complex.	Frontiers in Medicine . 2020;7:1.	Case report
**Malissen N**	Long-term treatment of cutaneous manifestations of tuberous sclerosis complex with topical 1% sirolimus cream: A prospective study of 25 patients.	Journal of the American Academy of Dermatology. 2017;77(3):464 -472.e463.	Case report
**Lee YI**	Comparative Effects of Topical 0.2% Sirolimus for Angiofibromas in Adults and Pediatric Patients with Tuberous Sclerosis Complex.	Dermatology.2018;234 (1-2):13-22.	Case report
**Krakowski AC**	Inhibition of angiofibromas in a tuberous sclerosis patient using topical timolol 0.5% gel.	Pediatrics.2015;136(3) e709-e713.	Case report
**Knopfel N**	Topical 0.2% rapamycin to treat facial angiofibromas and hypomelanotic macules in tuberous sclerosis.	Actas Dermo Sifiliograficas.2014;10 5(8):802-803.	Case report
**Hofbauer GF**	The mTOR inhibitor rapamycin significantly improves facial angiofibroma lesions in a patient with tuberous sclerosis.	British Journal of Dermatology.2008;159 (2):473-475.	Case report
**Hatano T**	Improved health-related quality of life in patients treated with topical sirolimus for facial angiofibroma associated with tuberous sclerosis complex.	Orphanet Journal Of Rare Diseases 2020;15(1):133.	Case report
**Ebrahimi-Fakhari D**	Topical Rapamycin for Facial Angiofibromas in a Child with Tuberous Sclerosis Complex (TSC): A Case Report and Long-Term Follow-up.	Dermatology and Therapy.2017;7(1):175 -179.	Case report

**Csoma ZR**	Successful treatment of facial angiofibromas with local sirolimus in childhood, report of two cases.	Pediatric Dermatology. 2019;36:S41-S42.	Case report
**Correa MC**	Topical sirolimus with satisfactory response in facial angiofibromas of tuberous sclerosis-Two case reports.	Journal of the American Academy of Dermatology. 2017;76(6):AB262.	Case report
**Bottyan K**	Successful treatment of facial angiofibromas with local sirolimus in childhood in Bourneville-Pringle disease.	Orvosi Hetilap. 2019;160(13):516-520.	Case report
**Batalla A**	Topical rapamycin and facial angiofibromas in tuberous sclerosis.	Piel. 2016;31(7):467-470.	Case report
**Balestri R**	Analysis of current data on the use of topical rapamycin in the treatment of facial angiofibromas in tuberous sclerosis complex.	J Eur Acad Dermatol Venereol. 2015;29(1):14-20.	Case report
**Amin S**	Novel treatment option of sirolimus ointment for facial angiofibromas in individuals with tuberous sclerosis complex (TSC).	Archives of Disease in Childhood. 2016;101:A143.	Case report
**Nct.**	Topical Rapamycin to Erase Angiofibromas in TSC.	https://clinicaltrialsgov/show/NCT01526356. 2012.	Insufficient data despite of request
**Jprn U.**	Randomized, double-blind, placebo-controlled, clinical trial with OSD-001 for skin lesions due to tuberous sclerosis complex.	http://wwwwhoint/trialsearch/Trial2aspx?TrialID=JPRNUMIN000012420. 2013.	Insufficient data despite of request
**Nct.**	Dose-Ranging Efficacy and Safety Study of Topical Rapamycin Cream for Facial Angiofibroma Associated With Tuberous Sclerosis Complex.	https://clinicaltrialsgov/show/NCT03826628. 2019.	Insufficient data despite of request
**Nct.**	Topical Sirolimus Ointment for Cutaneous Angiofibromas in Subjects With Tuberous Sclerosis Complex.	https://clinicaltrialsgov/show/NCT03363763. 2017.	Insufficient data despite of request
**Nct.**	Topical Rapamycin and Calcitriol for Angiofibroma of Tuberous Sclerosis..	https://clinicaltrialsgov/show/NCT03140449. 2017	Insufficient data despite of request
**Nct.**	Topical Everolimus in Patients With Tuberous Sclerosis Complex.	https://clinicaltrialsgov/show/NCT02860494. 2016.	Insufficient data despite of request
**Nct.**	Phase III Trial of Topical Formulation of	https://clinicaltrialsgov/show/NCT0	Insufficient data

	Sirolimus to Skin Lesions in Patients With Tuberous Sclerosis Complex (TSC).	2635789. 2015.	despite of request
**Nct.**	Topical Rapamycin Therapy to Alleviate Cutaneous Manifestations of Tuberous Sclerosis Complex (TSC) and Neurofibromatosis I (NF1).	https://clinicaltrialsgov/show/NCT01031901. 2009.	Insufficient data despite of request
**Jprn U.**	Clinical study of safety and efficacy of rapamycin topical medication for skin lesions due to tuberous sclerosis complex.	http://wwwwhoint/trialsearch/Trial2aspx?TrialID=JPRN-UMIN000015114. 2014.	Insufficient data despite of request
**Euctr FR.**	Topical Everolimus versus placebo for the treatment of facial angiofibromas in patients with tuberous sclerosis complex.	http://wwwwhoint/trialsearch/Trial2aspx?TrialID=EUCTR2018-002531-18-FR. 2018.	Insufficient data despite of request
**Euctr ES.**	Clinical trial in patients with tuberous sclerosis for the study of the effects of topical rapamycin in reducing facial tumors associated with the disease.	http://wwwwhoint/trialsearch/Trial2aspx?TrialID=EUCTR2011-006308-12-ES. 2012.	Insufficient data despite of request
**Koenig MK.**	Topical rapamycin therapy to alleviate the cutaneous manifestations of tuberous sclerosis complex: a double-blind,randomized, controlled trial to evaluate the safety and efficacy of topically applied rapamycin.	Drugs R D 2012;12:121-6.	No usable data
**Chen HL.**	Systematic Review and Network Meta-Analysis of Immune Checkpoint Inhibitors in Combination with Chemotherapy as a First-Line Therapy for Extensive-Stage Small Cell Carcinoma.	Stage Small Cell Carcinoma. Cancers (Basel) 2020;12.	No usable data

Table S3 Surface under the cumulative ranking curve (SUCRA) of clinical improvement

Treatment	SUCRA	Probability of being the best	Mean Rank
Placebo	0.0	0.0%	5.0
Topical sirolimus 0.05%	70.4	19.7%	2.2
Topical sirolimus 0.1%	28.6	0.0%	3.9
Topical sirolimus 0.2%	90.8	69.6%	1.4
Topical sirolimus 1%	60.2	10.7%	2.6

Table S4 Surface under the cumulative ranking curve (SUCRA) of treatments based on severe adverse events leading to withdrawal

Treatment	SUCRA	Probability of being the best	Mean Rank
Placebo	57.5	12.2%	2.7
Topical sirolimus 0.05%	47.4	39.4%	3.1
Topical sirolimus 0.1%	60.0	18.6%	2.6
Topical sirolimus 0.2%	53.5	23.4%	2.9
Topical sirolimus 1%	31.6	6.4%	3.7

Figure S1 Summary for risk of bias of included clinical trials. The green symbols represent low risk of bias, the yellow symbols represent unclear risk of bias, and the red symbols represent high risk of bias

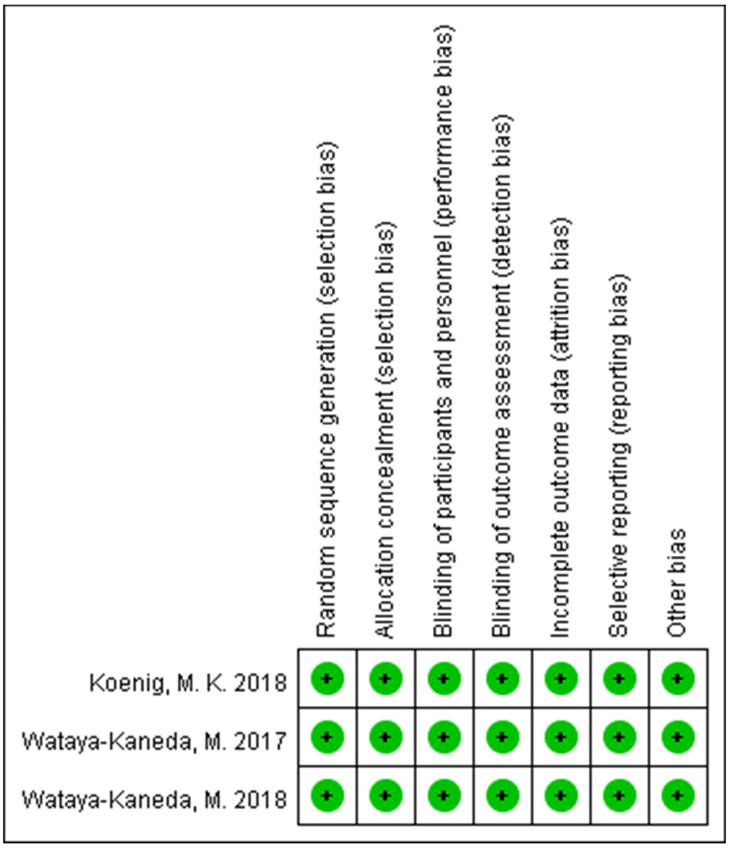

Figure S2 Forest plots of network meta-analysis of treatments for facial angiofibroma: analysis based on severe adverse events leading to withdrawal

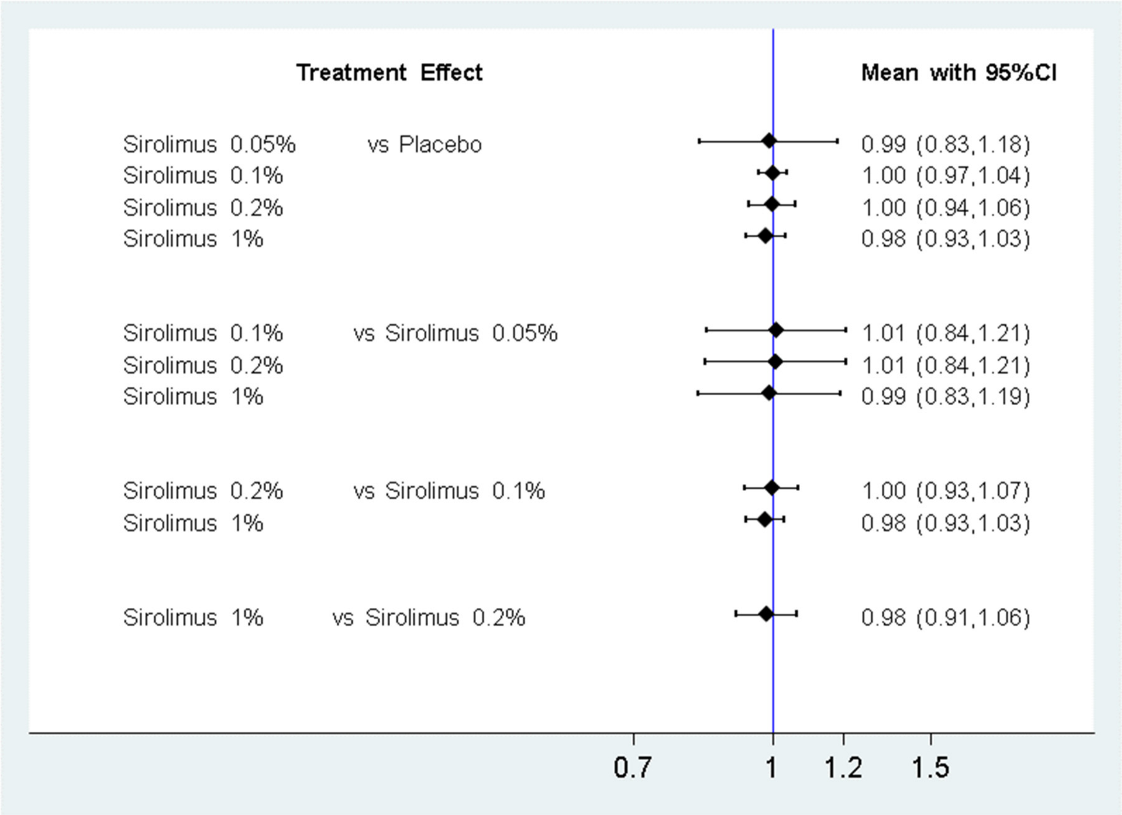

Figure S3 Clinical improvement

Forest plots from pairwise comparisons of treatment agents. The result of a single study was identified as direct evidence when pairwise meta-analysis for a specific comparison was not attainable.

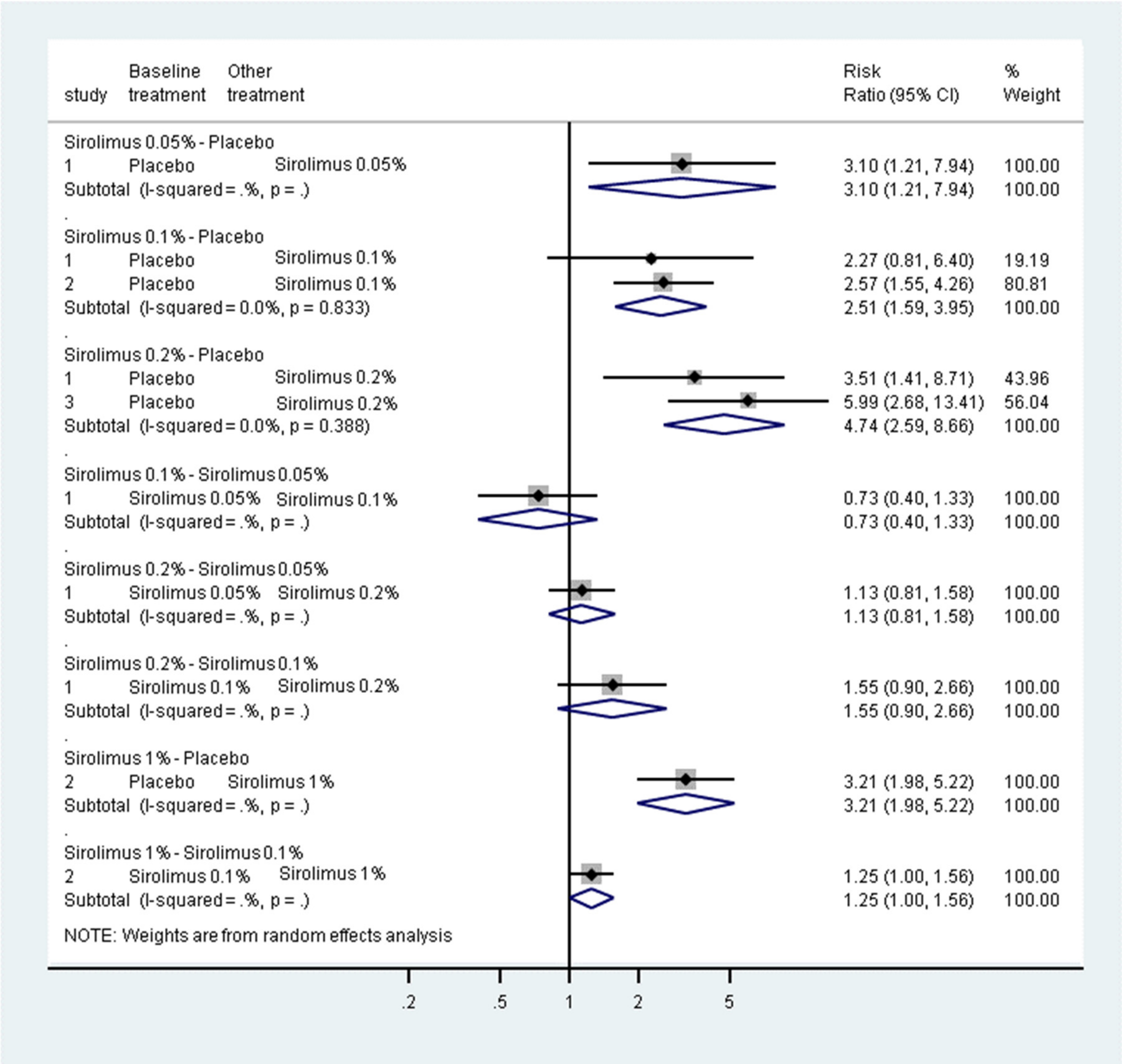

Figure S4 Severe adverse events leading to withdrawal

Forest plots from pairwise comparisons of treatment agents. The result of a single study was identified as direct evidence when pairwise meta-analysis for a specific comparison was not attainable.

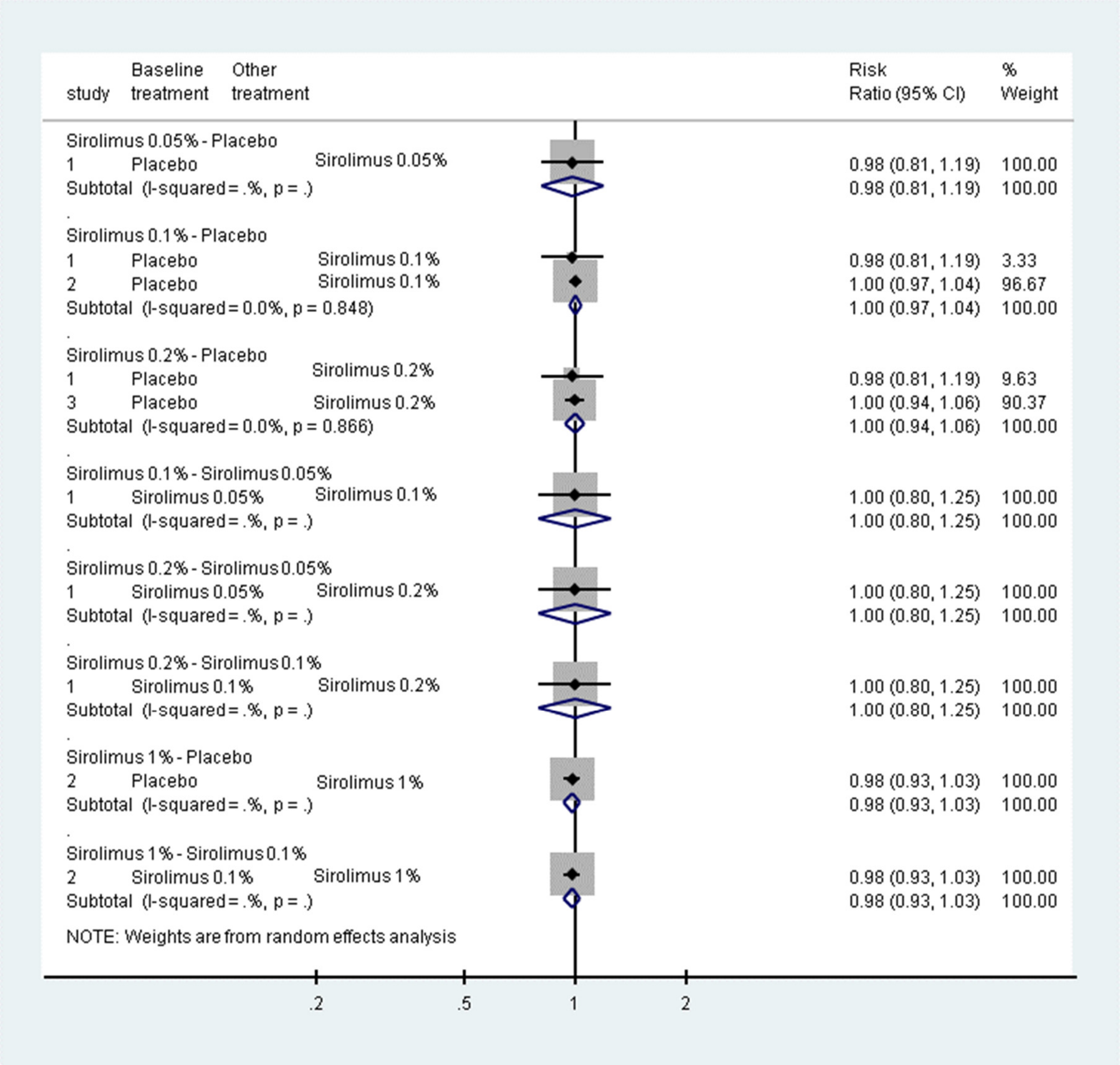

Supplement: Supplementary file 1 [file biomedicines-10-00826-s001.zip › biomedicines-1645691-Supplement.pdf]
